# Prevalence of breast cancer in rural population of Jaipur: a survey-based observational study

**DOI:** 10.1038/s41598-024-58717-0

**Published:** 2024-04-17

**Authors:** Roshni Singh, Sachin Kumar, Prashant Nakash, Ramesh Kumar, Govind Kumar, Pusparghya Pal, Shivang Mishra, Preeti Raj, Sumit Rajotiya, Anurag Kumar Singh, Sourav Debnath, Bhumi Chaturvedi, Hemant Bareth, Akhilesh Patel, Mahaveer Singh, Anurag Srivastava, Deepak Nathiya, Balvir Singh Tomar

**Affiliations:** 1 Department of Pharmacy Practice, Institute of Pharmacy, Nims University Rajasthan, Jaipur, India; 2 Department of Endocrinology, National Institute of Medical Sciences, Nims University Rajasthan, Jaipur, India; 3 Department of Surgical Disciplines & Breast Services, National Institute of Medical Sciences, Nims University Rajasthan, Jaipur, India; 4 Institute of Pediatric Gastroenterology and Hepatology, Nims University Rajasthan, Jaipur, India; 5 Department of Clinical Studies, Fourth Hospital of Yulin (Xingyuan), Yulin, Shaanxi China; 6 Department of Clinical Sciences, Shenmu Hospital, Shenmu, Shaanxi China

**Keywords:** Breast cancer, Prevalence, Physical screening, Rural, Cancer, Diseases, Oncology

## Abstract

Breast cancer, a global health concern predominantly affecting women, recorded 2.3 million new cases and 685,000 deaths in 2020. Alarmingly, projections suggest that by 2040, there could be over 3 million new cases and 1 million deaths. To assess breast cancer prevalence in 24 rural villages within a 60 km radius of NIMS Hospital, Tala Mod, Jaipur, Rajasthan, North India 303,121. A study involving 2023 participants conducted initial screenings, and positive cases underwent further tests, including ultrasound, mammography, and biopsy. SPSSv28 analysed collected data. Among 2023 subjects, 3 screened positive for breast lumps. Subsequent clinical examination and biopsy identified 1 normal case and 2 with breast cancer, resulting in a prevalence proportion of 0.0009 or 98 per 100,000. This study helps fill gap in breast cancer prevalence data for rural Rajasthan. The results highlight a concerning prevalence of breast cancer in the rural area near NIMS hospital, emphasizing the urgent need for increased awareness, early detection, and better healthcare access. Challenges like limited resources, awareness programs, and delayed diagnosis contribute to this high incidence. To address this, comprehensive approach is necessary, including improved screening programs and healthcare facilities in rural areas. Prioritizing rural healthcare and evidence-based strategies can reduce the burden of breast cancer and improve health outcomes.

## Introduction

Breast cancer is characterized by the unregulated proliferation and division of aberrant cells within the mammary gland. These cells may form lump or appear as visible abnormalities on mammogram. While breast cancer can affect both men and women, it is significantly more common in women. In 2020, the World Health Organization (WHO) reported 2.3 million breast cancer diagnoses and 685,000 deaths worldwide. By the end of 2020, there were 7.8 million women who had been previously diagnosed with breast cancer within the past five years and were currently alive, establishing it as the most widespread type of cancer worldwide^1^.

A research conducted by the International Agency for Research on Cancer (IARC) and its partner institutions predicts the future impact of breast cancer in 2040, drawing on the burden observed in 2020. It estimates that by 2040, there will be over 3 million new cases per year, representing a 40% increase, and more than 1 million deaths, indicating a 50% increase. This study was published in "The Breast"^2^.

In 1994, a study conducted by the Cancer Registry at Sawai Man Singh (SMS) Medical College in Jaipur recorded 2509 histologically proven cancer cases from various government and private hospitals in urban Jaipur. Among these cases, 19.4% were females with breast cancer, making it one of the prevalent types of cancer^3^.

Epidemiological studies have identified various factors that are associated with the onset and advancement of breast cancer. Risk factors such as late marriage, delayed first childbirth, and late menopause have been strongly linked to the incidence of the disease. Late marriage and childbirth can result in inadequate differentiation of breast tissue, increased exposure to non-estrogenic mutagens, and genotoxicity caused by estrogen^4^. Delayed menopause can lead to prolonged estrogen exposure. Conversely, early pregnancy and extended breastfeeding duration have decreased the risk of estrogen receptor-positive and estrogen receptor-negative breast cancer^5,6^.

There is a common misconception that breast cancer affects only women. However, men can also develop this disease, although it occurs in small numbers. According to the Centers for Disease Control and Prevention (CDC) in the United States, 1 out of every 100 diagnosed breast cancers is found in men^2^.

Early detection through screening programs and diagnostic tests is crucial to reduce breast cancer incidence and mortality. Several methods are available for breast cancer control, including Molecular Testing, Next-Generation Sequencing, Liquid Biopsy, Genetic Testing, and Artificial Intelligence^4^.

In this study, we aim to determine the prevalence of breast cancer in the rural population of Jaipur, Rajasthan, North India. This survey-based study was conducted approximately 60 kms from the NIMS Hospital, Tala Mod, Jaipur, Rajasthan, North India 303121.

## Methods

### Study design and population

This cross-sectional study was aimed to determine the prevalence of breast cancer. This study was conducted on 2023 participants who live in the rural areas of Jaipur, Rajasthan, within a radius of 60 kms from NIMS hospital, Tala Mod, Jaipur, Rajasthan, North India 303121. The study duration was 6 months, from October 2022 to March 2023.

Participants above 18 years and who fulfilled the inclusion criteria were screened and enrolled in the study, the participants who did not willingly participate or give their consent were excluded.

### Study recruitment procedure

After screening 2442 participants through the inclusion and exclusion criteria, total no of 2023 participants were enrolled from 24 rural villages of Jaipur, including Basna, Nimbi, Kalwad, Achrol, Jhotwara, Manoharpur, Khojawala, Shahpura, Beelpur, Majipura, Dhand, Harwar, Peelwa, Lakher, Noorpur, Bhuranpura, Tala, Gunawata, Bhikhanwala, Chharsa, Chandawas, Dhaler, Syari, Bilonchi.

### Data collection

A data collection form was designed for physical screening, which includes demographic details like participants' age, gender, social history like residential area, occupation, marital status, smoking, and alcohol status. Female participants were interviewed for their age at menarche, age at first childbirth, number of children, and history of breastfeeding. A detailed examination of the breast was done to see the symmetry of the breast, skin changes, retraction, tenderness, nipple retraction, lymph node, lump, consistency, and mobility of the lump using a breast cancer screening data collection form^9^.

### Clinical investigation

The final identification of the cancerous site was done through mammography and ultrasonography using Mammography System MAM-VENUS, ALLENGERS Medical Systems Ltd. Sector-34, Chandigarh, India, and E-CUBE 5 ULTRASOUND IMAGING SYSTEM ALPINIONO MEDICAL SYSTEMS CO., Ltd. Seoul Republic of Korea, 19-06-2018 respectively^7,8^. Biopsy was performed to have final diagnosis of breast cancer in participants with positive physical screening as per American Society of Clinical Oncology (ASCO) guidelines^10^.

### Ethics

In accordance with the ethical principles outlined in the Declaration of Helsinki, the Institutional Review Board of NIMS University Rajasthan, Jaipur, granted clearance for the current study to proceed (approval number: NIMSUR/IEC/2022/349). Informed consent was taken from all the participants.

### Statistical analysis

The IBM SPSS version 28.0 programme was used to analyse the data, and Excel version 2019. Descriptive statistical methods were used to encapsulate the data: continuous variables were presented using the standard deviation, mean, median, and category variables were expressed in frequency and proportion. The prevalence rate and prevalence proportion were calculated using the standard formula^11^.

## Results

In total, 2023 participants were enrolled in this study from 24 different rural villages of Jaipur, Rajasthan, India. Out of which 1088 were females. Participants above 18 years had a mean age of 43.79 ± 14.7 years. In total, 1815 (89.72%) were married. The occupations of the participants were classified according to Kuppuswamy's classification. These included professionals 133 (6.57%), semi-professionals 19 (0.94%), shop/farmers 820 (40.53%), skilled workers 65 (3.21%), semi-skilled workers 5 (0.25%), unskilled worker 35 (1.73%), and unemployed 946 (46.76%). Among them, 304 (15.03%) were alcoholics, and 700 (34.60%) were smokers, as seen in Table 1. Among female participants, the mean age of menarche was 12.82 ± 1.039, and the mean age at first childbirth was 20.8 ± 2.461, having a median of 3[2–4] children as seen in Table 1.

**Table 1:** Descriptive data of all the enrolled patients.

Variables	n
Total participant, n	2023
Total Breast cancer patients, n (%)	2 (0.09)
Total patient with lump, n (%)	3 (0.14)
Age (mean ± SD)	43.79 ± 14.77
Gender, n (%)	
Male	935 (46.2)
Female	1088 (53.8)
Marital status, n (%)	
Married	1815 (89.72)
Unmarried	208 (10.28)
Occupation, n (%)	
Professional	133 (6.57)
Semi professional	19 (0.94)
Shop/farmer	820 (40.53)
Skilled worker	65 (3.21)
Semi-skilled worker	5 (0.25)
Unskilled worker	35 (1.73)
Unemployed	946 (46.76)
Alcohol (n %)	
Yes	304 (15.03)
No	1719 (84.98)
Smoking, n (%)	
Yes	700 (34.60)
No	1323 (65.40)
Age of Menarche (mean ± SD)	12.82 ± 1.039
Age at first childbirth in year (mean ± SD)	20.8 ± 2.461
Number of children [IQR(Q1–Q3)]	3[2–4]
1 to 3, n (%)	595 (61.40)
4 to 6, n (%)	344 (35.50)
7 to 9, n (%)	30 (3.10)
Physical examination, n (%)	
Symmetry in breast shape	2021 (99.9)
Skin change	1 (0.01)
Breast retraction	1 (0.01)
Nipple retraction	1 (0.01)
Breast tenderness	1 (0.01)
Lump	3 (0.1)
Prevalence (breast cancer)	0.09%
Prevalence Proportion (breast cancer)	0.0009

The clinical examination of 3 subjects with positive physical screening is presented in Table 2. Subjects 1, 2, and 3 were 23, 44 and 50 years respectively. Subjects 1 and 3 were homemakers, subject 2 was farmer, and all three subjects were married. The age of menarche of subject 1, 2, and 3 was 12, 14, and 13 years, respectively, and the age of their first childbirth were 22, 18, and 22 years, respectively, and they had 1, 2, and 3 no. of children, respectively. Subjects 2 and 3 had history of breastfeeding, while subject 1 did not. Subjects 1 and 3 had symmetry in their breast shape, while the breast shape of subject 2 was asymmetric. The skin change was seen in subject 2, and the retraction in the breast and nipple was seen in subject 3, while the lymph node was enlarged in subject 2.

**Table 2:** Clinical examination of patients with positive physical screening.

Parameters	Subject 1	Subject 2	Subject 3	Prevalence Proportion
Age	23	44	50	
Gender	Female	Female	Female	
Occupation	Housewife	Farmer	Housewife	
Marital status	Married	Married	Married	
Diet	Vegetarian	Vegetarian	Vegetarian	
Smoking	No	No	No	
Alcohol	No	No	No	
Age of menarche	12	14	13	
Age of first childbirth	22	18	22	
Number of children	1	3	2	
History of breast feeding the children	No	Yes	Yes	
Symmetry in breast shape	Yes	No	Yes	0.0009
Skin change	No	Yes	No	0.0005
Retraction	No retraction	No retraction	Yes	0.0005
Nipple retraction/distortion	No	No	Yes	0.0005
Lymph node	Not enlarged	Enlarged	Not enlarged	0.0005
Supra clavicular nodes	Not enlarged	Not enlarged	Not enlarged (pain)	
Infra clavicular nodes	Not enlarged	Not enlarged	Not enlarged (pain)	
Breast tenderness	Absent	Absent	Present	0.0005
Lump	Present	Present	Present	0.0015
Lump position if present	Right	Right	Right	
Lump size(cm) if present		2.4*1.8 cm	0.3*0.2 cm	
Consistency	–	Hard	Soft	
Mobility of the lump	–	Fixed to the skin	Fixed to chest wall	
Clinical breast examination result	Normal	Cancer	Cancer	0.0009

Of the 2023 participants physically screened, lump was found in 3 females, of which 2 were confirmed as breast cancer, yielding prevalence proportion of 2 (0.00098), and prevalence was 0.09%, which determined the prevalence rate 98 per 100,000 population.

## Discussion

In India, survival of breast cancer after five years of diagnosis ranges to 66%. Epidemiological studies indicate that the worldwide burden of breast cancer is projected to exceed nearly 2 million cases by the year 2030^1^. Our study reports the prevalence of breast cancer in rural populations around 60 km radius of NIMS hospital. This study comprises 2023 participants who satisfied the predetermined inclusion and exclusion criteria and were enrolled. Among these participants, 46.21% were male and 53.78% were female.

During the survey, participants from 24 rural villages were included; the age cut-off for the screening population was above 18 years. As per the CDC, the age threshold for diagnosis is over 50 years. Still, due to the increased prevalence of breast cancer among the young population, the bar has been lowered to 18 years^12^.

Out of 2023 study subjects, 3 were screened with positive criteria in pre-screening, and 2 were tested positive with breast cancer yield prevalence proportion of 0.0009. Later, the screening-positive patients were invited to the surgery outpatient department (OPD) of NIMS Hospital for further clinical examination. After considering all relevant parameters and reviewing the biopsy reports, a definitive diagnosis was established, categorizing the subjects into one of the following groups: cancer, benign, and normal.

Of the 3 positive subjects, the first was female and diagnosed normal in breast biopsy.

The second subject was a 44-year married female, age of menarche was 14 years and had 3 children. Enlarged lymph nodes in the right breast with a size of 2.4 × 1.8 cm with hard consistency were diagnosed. The third subject was a 50-year-old married female with 2 children. An asymmetrical, retracted breast with nipple discharge and a lump in the right breast of size 0.3 × 0.2 cm were diagnosed. Both the diagnosed subjects were above the age of 40.

To date, few studies have been conducted in India reporting breast cancer screening. The study conducted by Neethu et al. on community-engaged cancer focuses on the engagement of breast cancer and implementing a comprehensive cancer screening strategy; another retrospective study by Deepti et al. on breast cancer in young women. However, any of these studies do not include the prevalence of breast cancer in the rural population of Rajasthan, India. Thus, this door-to-door cross-sectional study reports the prevalence of breast cancer in the rural population of Rajasthan, India, reporting the 0.0009% prevalence of breast cancer^13^.

### Limitations

Limited funding restricted us from advancing our survey-based study to more villages. Additionally, the lack of awareness-based programs and social stigmas about breast cancer in rural populations posed challenges during data collection.

## Conclusion

In conclusion, the prevalence of breast cancer in the 60 km radius of NIMS hospital is a significant concern. The study has shed light on the alarming rate of breast cancer, emphasizing the need for increasing awareness, early direction, and improving access to health care services. In this study, the prevalence of breast cancer was found to be 0.0009 around 60 km of NIMS hospital covering 24 villages. Limited health resources, lack of awareness programs, and delayed diagnosis increase the risk of breast cancer. Addressing these challenges required multifaceted approaches, improving screening programs, and establishing comprehensive healthcare facilities. By investing in these initiatives and prioritizing the well-being of individuals residing in rural areas, we can work towards reducing the burden of breast cancer and improving overall health outcomes for these communities. Government authorities must implement evidence-based strategies to ensure that rural areas receive the necessary resources and support to combat breast cancer effectively.

## Data Availability

The study incorporates the original contributions, and for additional inquiries, please contact the corresponding authors.

## Abbreviations

WHO: World Health Organization
IARC: International Agency for Research on Cancer
S.M.S: Sawai Man Singh
CDC: Centers for Disease Control and Prevention
ASCO: American Society of Clinical Oncology
OPD: Outpatient department
